# Selection of an Appropriate Protein Extraction Method to Study the Phosphoproteome of Maize Photosynthetic Tissue

**DOI:** 10.1371/journal.pone.0164387

**Published:** 2016-10-11

**Authors:** Inês M. Luís, Bruno M. Alexandre, M. Margarida Oliveira, Isabel A. Abreu

**Affiliations:** 1 Instituto de Tecnologia Química e Biológica António Xavier, Universidade Nova de Lisboa (ITQB-UNL), Oeiras, Portugal; 2 Instituto Biologia Experimental e Tecnológica (iBET), Oeiras, Portugal; Henan Agricultural University, CHINA

## Abstract

Often plant tissues are recalcitrant and, due to that, methods relying on protein precipitation, such as TCA/acetone precipitation and phenol extraction, are usually the methods of choice for protein extraction in plant proteomic studies. However, the addition of precipitation steps to protein extraction methods may negatively impact protein recovery, due to problems associated with protein re-solubilization. Moreover, we show that when working with non-recalcitrant plant tissues, such as young maize leaves, protein extraction methods with precipitation steps compromise the maintenance of some labile post-translational modifications (PTMs), such as phosphorylation. Therefore, a critical issue when studying PTMs in plant proteins is to ensure that the protein extraction method is the most appropriate, both at qualitative and quantitative levels. In this work, we compared five methods for protein extraction of the C4-photosynthesis related proteins, in the tip of fully expanded third-leaves. These included: TCA/Acetone Precipitation; Phenol Extraction; TCA/Acetone Precipitation followed by Phenol Extraction; direct extraction in Lysis Buffer (a urea-based buffer); and direct extraction in Lysis Buffer followed by Cleanup with a commercial kit. Protein extraction in Lysis Buffer performed better in comparison to the other methods. It gave one of the highest protein yields, good coverage of the extracted proteome and phosphoproteome, high reproducibility, and little protein degradation. This was also the easiest and fastest method, warranting minimal sample handling. We also show that this method is adequate for the successful extraction of key enzymes of the C4-photosynthetic metabolism, such as PEPC, PPDK, PEPCK, and NADP-ME. This was confirmed by MALDI-TOF/TOF MS analysis of excised spots of 2DE analyses of the extracted protein pools. Staining for phosphorylated proteins in 2DE revealed the presence of several phosphorylated isoforms of PEPC, PPDK, and PEPCK.

## Introduction

One of the critical steps in a proteomics study is the selection of the most appropriate protein extraction method. The extraction method must take into consideration the nature of the sample and the biological question to be addressed. For differential proteomic analysis and protein isoform characterization, it is crucial that the protein extraction method allows the preservation of protein post-translational modifications (PTMs). In plant proteomics, this challenge is raised to another level as plant tissues may contain high amounts of polyphenols, lipids, and polysaccharides, which are known to interfere with protein separation, and may induce modifications in the protein amino acid residues [1]. Thus, it is common to use two major protein extraction methods to remove these interfering compounds. The methods commonly used always rely on protein precipitation steps and include precipitation with TCA/acetone and phenol extraction either, alone or in combination [1, 2]. Protein precipitation can rapidly promote protease inhibition, which can be useful. On the other hand, difficulties with the re-solubilization of proteins after precipitation can lead to incomplete protein profiles and the organic solvents used during the extraction can lead to the loss of some labile PTMs, thus compromising phosphoprotein extraction. The protein extraction in a urea-based denaturing buffer can also promote protease inhibition without any need for protein precipitation [2]. This type of extraction is, however, generally considered unsuitable for plant material and it is rarely used in plant proteomics, even though some plant materials (*e*.*g*.: plantlets and young leaves) are easy to extract. Recently, Wu *et al*. [3] compared the classical precipitation-based protein extraction methods with direct cell lysis in a urea-based buffer. The authors used the 5^th^ maize leaf as the youngest tissue and concluded that the combination of TCA/acetone precipitation with a phenol extraction was an all-purpose sample preparation method, however, phosphoproteome conservation was not analyzed.

For the identification of new phosphorylation sites in C4-photosynthesis key enzymes, the ideal protein extraction method should ensure good protein recovery with minimum phosphorylation loss. The tip of the fully expanded third maize leaf is a photosynthetic tissue with an active C4-photosynthetic apparatus [4, 5]. So, we hypothesized that since these leaves are young tissues and should not be rich in interfering substances, they can be processed using a urea-based buffer (Lysis Buffer—LB) to avoid phosphoprotein losses due to a precipitation step.

**Fig 1:**
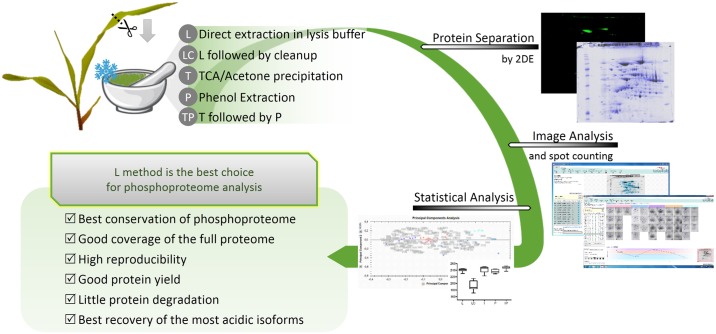
Overview of the experimental design and main conclusions. Maize leaves were ground to powder in liquid nitrogen. Proteins were extracted by five extraction methods: direct extraction in Lysis Buffer (L); L followed by Cleanup with a commercially available kit (LC); TCA/Acetone Precipitation (T); Phenol Extraction (P); T followed by P (TP). Protein extracts were separated by 2DE and stained with a phosphoproteome specific stain and with a whole proteome stain. Gel images were acquired to evaluate the performance of each extraction method and determine the statistical significance of the differences observed. L was revealed as the best choice for phosphoproteome analysis.

Gel-free proteomics strategies have become increasingly popular due to the emergence of sophisticated mass spectrometers. Nevertheless, proteomics workflows relying on two-dimensional gel electrophoresis (2DE) that separate intact proteins based on their isoelectric point and molecular weight, are still amongst the most robust and reproducible proteomics platforms. Moreover, 2DE gel-based proteomics is a very useful approach to study protein PTMs, since many modifications induce shifts in pI or molecular weight, or in both [6]. In this sense, 2DE enables separation between protein isoforms that differ only in the PTMs they carry. Furthermore, the use of multi-staining approaches on a single gel can improve 2DE resolving power. For example, “Coomassie Brilliant Blue” (CBB) staining allows assessment of the total protein abundance, while “Pro-Q Diamond Phosphoprotein Stain” stains phosphoproteins (Pro-Q diamond, or PQD, from this point on, can bind to the phosphate moiety of the phosphorylated residue directly, regardless of its nature) [7]. Both staining techniques on the same 2DE gel allow identification of the subset of proteins in the visible proteome that is composed by phosphoproteins. We used 2DE combined with CBB and PQD stains to evaluate the differences in protein profiles obtained from five tested protein extraction methods, see Fig 1 for an overview. This allowed us to select the most appropriate method to study protein phosphorylation in maize C4-photosynthesis proteins. We report the direct extraction in Lysis Buffer as the method of choice to study young photosynthetic maize leaf phosphoproteome, avoiding the precipitation steps of conventional methods. We show the applicability of this method for the analysis of proteins by 2DE gels while providing a 2DE map of the extracted proteome, after protein identification in an MALDI-TOF/TOF mass analyzer. The 2DE-MS approach presented here allowed the mapping of most of the main proteins involved in the C4-photosynthesis metabolism and several of their isoforms. The co-localization of CBB and PQD spots offer insight on the phosphorylation state of some of the identified proteins.

## Material and Methods

### Reagents

All reagents used in this study are analytical or HPLC/MS grade. PhosStop, cOmplete EDTA-free, and pepstatin were acquired from Roche Applied Science (Germany). Nuclease Mix, Destreak Reagent, IPG Buffer pH 4–7, and 7 cm Immobiline^®^ Drystrips pH 4–7 were obtained from GE Healthcare (UK). The phosphoprotein stain Pro-Q^®^ Diamond (PQD) and the PeppermintStick^™^ phosphoprotein molecular weight markers were purchased from Life Technologies (CA, USA). The whole proteome Coomassie Brilliant Blue stain, BlueSafe (CBB) and the protein molecular weight markers NZYColour Protein Marker II were acquired from NZYTech (Portugal). Porcine trypsin was acquired from Promega Corporation (WI, USA). Seeds from *Zea mays* inbred line B73 used in this study were amplified in our greenhouse over the years. Original seeds were kindly provided by Dr. Christoph Peterhansel (University of Hannover, Germany).

### Maize Growth

*Zea mays* inbred line B73 seeds were germinated, and plantlets were transferred to soil:turf (1:1) mixture. Plants were grown with 70% humidity, in light/dark cycles of 16/8 h at 28°C/26°C, with a light intensity of 450 μmol m^-2^ s^-1^. One-third (from leaf tip) of the maize 3^rd^ leaves were harvested from 12 day-old plants and immediately frozen in liquid nitrogen.

### Sample Preparation

Maize-leaf tissue was ground to a fine powder with mortar and pestle in liquid nitrogen and lyophilized. Twelve tubes, each one with an equivalent of 230 mg of fresh weight (FW), corresponding to 30 mg of dry powder, were prepared and used to extract protein in triplicate, using five different methods. These methods were: direct extraction in Lysis Buffer (L); direct extraction in Lysis Buffer followed by Cleanup using a commercially available kit (LC); TCA/Acetone Precipitation (T); Phenol Extraction (P); and TCA/Acetone Precipitation followed by Phenol Extraction (TP). Extraction methods were performed as described below:

L: Powder aliquots were lysed in Lysis Buffer (LB) [7M Urea, 2M Thiourea, 30mM Tris, 4% (w/v) CHAPS, 4% (v/v), cOmplete, EDTA-Free 25x, 0.1% (v/v) Pepstatin 1mM, 1% (v/v) Nuclease Mix 100x, 10% PhosSTOP 10x] in the proportion 0.5 g of FW to 1 mL of LB. Tubes were homogenized and allowed to stay on ice for 30 min and then were subjected to several subsequent centrifugations (4°C, 17,000 x *g*, 30 min) to remove debris.

LC: Aliquots of the samples obtained in method L were processed using 2-D Clean-Up Kit (GE Healthcare, UK) according to the manufacturer’s instructions and the obtained pellet was re-suspended in new LB, as described above for samples L.

T: Powder was directly precipitated in TCA/acetone following the procedure described by Méchin [8] with slight modifications: we used DTT as reducing agent, and each powder aliquot was precipitated with 1 mL of TCA/acetone (1:9) supplemented with 20 mM DTT. The pellet obtained in this method was solubilized in LB, as described above for L samples.

P: Proteins were phenol extracted and then precipitated with ammonium acetate/methanol following the procedure outlined by Faurobert [9], with significant differences at the level of the extraction buffers and centrifugations. Proteins were first solubilized with SDS Extraction buffer (SEB) [1% (w/v) SDS, 150 mM Tris-HCl pH 8.8, 20 mM DTT, 1 mM EDTA, 0.1% (v/v) Pepstatin 1 mM, 4% (v/v) cOmplete, EDTA-Free 25x] in a powder FW/SEB ratio of 1:3. Phenol back extraction was performed using 10 mM Tris-HCl (pH 8.0), 1 mM EDTA, 0.7 M Sucrose and the final pellet was resuspended in LB, as described above. The phenolic phase was recovered by centrifugation at 16,160 x *g* (Room Temperature (RT), 10 min). After this, protein was precipitated with cooled 0.1 M Ammonium Acetate/Methanol, and the pellet was recovered by centrifugation at 15,000 x *g* (4°C, 10 min).

TP: Proteins were first precipitated with TCA/acetone applied directly to the plant powder, as described for T samples. The resulting pellet was then resuspended in SEB and protein extraction was carried out as described for P samples. The pellet obtained after phenol extraction was re-suspended in LB as described above for L samples.

Protein quantification was performed with 2-D Quant kit (GE Healthcare, UK) following the manufacturer’s instructions for all methods.

### 2DE Separation

Seven centimeter Immobiline DryStrip gels (IPG strips) with linear pH 4–7 were passively rehydrated overnight with 125 μL of Rehydration Buffer (RB) [7M Urea, 2M Thiourea, 30 mM Tris, 4% (w/v) CHAPS], supplemented with 1.6% (v/v) Destreak Reagent. Eighty micrograms of total protein were diluted to a final volume of 100 μL by adding RB with 1.6% (v/v) IPG Buffer pH 4–7 and 20 mM DTT, and loaded by cup at the anode. Isoelectric focusing (IEF) was performed on Ettan IPGphor 3 (GE Healthcare, UK) at 20°C, with a total 16,000 V.h using a 5-step program. The program consisted of two subsequent linear gradients to 300 V and 500 V for 1.5 h and 3 h, respectively, 0.5 h at a constant voltage of 500 V, a new linear gradient to 2,000 V for 1h and finally 6.5 h at a constant voltage of 2,000 V.

After IEF, IPG strips were equilibrated in Equilibration Buffer (EB) [6M Urea, 1.5M Tris-HCl pH 8.8, 30% (v/v) Glycerol, 2% (w/v) SDS] supplemented with 1% (w/v) DTT and then in EB supplemented with 2.5% (w/v) iodoacetamide (IAA). Equilibrated strips were loaded on top of a 10% Tris-HCl SDS-polyacrylamide gel together with two molecular weight standards loaded in two pieces of 3M paper: 3 μL of NZYColour Protein Marker II alongside pH 4; and 1 μL of PeppermintStick Phosphoprotein Molecular Weight Standards alongside pH 7. Both the IPG strip and molecular weight standards were fixed against the resolving gel with 1% (w/v) low melting agarose prepared in TGS buffer [25 mM Tris, 192 mM Glycine, 0,1% (w/v) SDS]. The second dimension was run with TGS buffer on a Mini-PROTEAN Tetra Cell (Bio-Rad Laboratories, CA, USA) at a constant voltage, starting with 25 V for 15 min and then 200 V until the bromophenol blue line reached the bottom of the gel. For each sample prepared, 2DE maps were prepared in triplicate.

### Gel staining, image acquisition and analysis

Gels were first stained with Pro-Q Diamond phosphoprotein stain (PQD) as previously described by Agrawal [10]. Images were acquired on FLA 5100 (Fujifilm Holdings Corporation, Japan) with a 532 nm laser using the DGR1 (270DF20) filter with a resolution of 25 μm, and were normalized using the PeppermintStick^™^ phosphoprotein molecular weight markers according to the manufacturers’ instructions. After PQD image acquisition, gels were directly stained with BlueSafe (CBB), according to the manufacturer’s instructions, and a gel image was obtained on an ImageScanner (GE Healthcare, UK). Gel images were analyzed with Samespots software version 4.6 (TotalLab, UK), to compute normalized volumes for each spot and assess their differential abundance using an ANOVA test. Spot counting was performed by manually inspecting the presence or absence of each spot in all gels. Statistical analysis of the total number of spots in each condition was performed by the Mann-Whitney U test. For the generation of the Venn diagrams, spots were considered to be present when observed in at least 50% of the gels.

### Protein Identification

Proteins were excised from 2DE gels stained with CBB. Gel pieces were destained with 50% Acetonitrile (ACN) in 50 mM Ammonium Bicarbonate (AmB). After destaining, the gel pieces were dehydrated with 100% ACN and proteins were reduced in 10 mM DTT in 25 mM AmB at 56°C for 45 min and alkylated using 55 mM IAA in 20 mM AmB at RT in the dark for 30 min. Gel pieces were dehydrated again, and trypsin (1:20 trypsin to protein ratio) was allowed to enter the gel pieces (45 min, 4°C). Trypsin excess was removed before proteins were digested for 12 h at 37°C in 50 mM AmB. Tryptic digests were collected and dehydrated in Speed Vac. Peptides were re-suspended in 0.1% trifluoroacetic acid (TFA), desalted in C18 Zip-Tips (EMD Millipore, MA, USA) and spotted on the MALDI plate using α-cyano-4-hydroxycinnamic acid as a matrix.

Analysis by mass spectrometry (MS) was performed at the iBET/ITQB-UNL MS unit (UniMS, Oeiras, Portugal) on a 4800plus MALDI-TOF/TOF (AB Sciex, Washington, DC, USA). The mass spectrometer was operated in reflector and positive ion modes. After MS acquisition, the top-20 most abundant ions were automatically selected for MS/MS data acquisition. Database searches were performed with ProteinPilot V4.5 (AB Sciex, Washington, DC, USA) using Mascot (Matrix Science, UK). Iterative searches were conducted to allow less constrained taxonomic restriction and increased precursor mass tolerance to achieve protein identification on the spots with no identification with the previous search parameters set. All searches were performed with these fixed parameters: MS/MS fragment tolerance set on 0.3 Da, 2 missed cleavages allowed, and carbamidomethylation on the Cys residues and oxidation of Met residues set as a fixed and variable modification, respectively. In a first search, mass tolerance was set to 50 ppm as the Swiss-Prot database (548208 sequences, 195282524 residues, downloaded in April 2015) was searched with taxonomic restriction to *Zea mays* while, in a second search, the mass tolerance was also set to 50 ppm, but with a taxonomic restriction for *Viridiplantae*. In a third search, mass tolerance was set to 100 ppm with no taxonomy restriction using the same database. Protein identification was considered positive whenever the MOWSE score was above the threshold defined for each search. Searches including the phosphorylation of Thr, Ser, and Tyr as variable modifications were also performed, but no phosphorylated peptides were identified.

## Results and Discussion

### Comparison of protein extraction methods

Five protein extraction methods were tested using third maize leaves to evaluate their ability to recover phosphorylated proteins, while ensuring protein integrity. The methods tested were: direct extraction in Lysis Buffer (L); L followed by Cleanup using a commercial kit (LC); TCA/Acetone Precipitation (T); Phenol Extraction (P); and TCA/Acetone Precipitation followed by Phenol Extraction (TP). Except for P, all other methods allowed the extraction of protein with comparable yields (Table 1), with the L and T methods resulting in the highest yields.

**Table 1:** Protein yield in μg of protein per mg of fresh weight, obtained by the five methods tested.

Extraction method	Yield (μg protein/ mg of fresh weight)
L	11,93 ± 1,69
LC	9,81 ± 0,34
T	12,09 ± 0,56
P	3,60 ± 0,87
TP	7,60 ± 0,42

**Fig 2:**
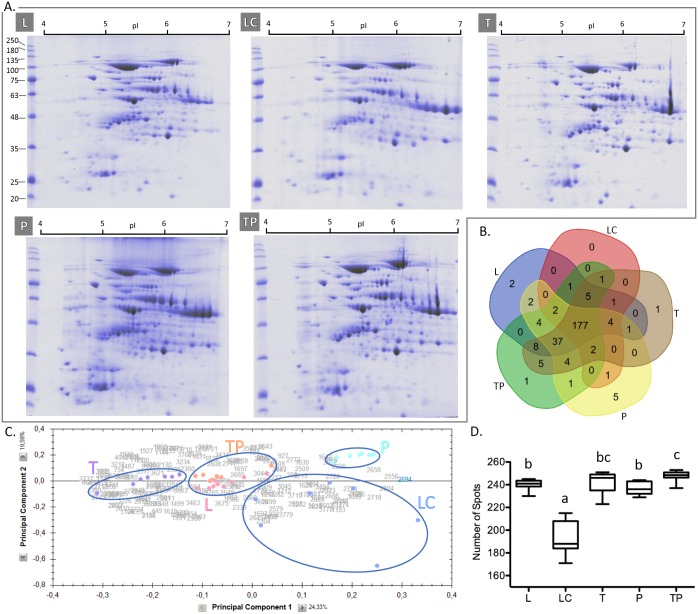
Comparative study of proteomes obtained using five different extraction methods. A: 2DE gels of protein extracts obtained with the extraction methods: L—Lysis Buffer, LC—Lysis Buffer + Cleanup, T—TCA/Acetone Precipitation, P—Phenol Extraction, TP—TCA/Acetone Precipitation + Phenol Extraction stained with Coomassie brilliant blue. B: Venn diagram summarizing the overlap of the total 265 spots detected in whole proteome analysis. C: Principal component analysis of the 190 spots found to have different abundances within the five methods: L (pink); LC (blue); T (purple); P (turquoise blue); TP (orange). D. Box plot of the total number of detected spots; different letters indicate that the spot counting was statistically different (p < 0.05) between the extraction methods. Mw markers: molecular weight markers in kDa; pI: isoelectric point.

The results obtained for the whole proteome analysis in 2DE gels stained with Coomassie brilliant blue (CBB) showed that all the methods tested produced well-resolved protein maps, with no apparent protein degradation (Fig 2A). Furthermore, all methods allowed good separation during IEF. This behavior probably results from the fact that third maize leaves are a non-recalcitrant tissue that contain small amounts of interfering substances. These non-recalcitrant properties are a key feature to achieve high-quality 2DE maps when methods that do not rely on a protein purification step are used to extract protein, such as the method L described in this work.

In whole proteome analysis, we detected a total of 265 spots. After comparing the five methods based on the median value, the TP method exhibited the highest number of protein spots (248.5 spots). For L, LC, T, and P methods, the median of the number of detected spots were 241, 188, 246, and 236, respectively (Fig 2D). The statistical analysis revealed that the number of detected spots in the LC method was statistically different (p < 0.05) from the other methods. The other methods all have a similar number of spots, and only the differences between TP and L and TP and P are statistically significant (p < 0.05) (Fig 2D). Among the tested methods, 190 of the detected protein spots were found to be differently abundant (p < 0.05). Principal component analysis (PCA) (Fig 2C) of the differently abundant spots shows a clear separation between the different methods, with an exception for L and TP that group together. This PCA distribution means that different extraction methods result in different profiles of extracted proteins. The grouping of L and TP methods shows the similarity of the protein profiles obtained with these two methods. When analyzed together, the PCA analysis and the box plot (Fig 2C and 2D) show that the LC method is the least efficient and reproducible.

As reported previously by Wu *et al*. [3], the combination of TCA/acetone followed by phenol extraction (a TP-like method) generates the highest number of protein spots. The authors also reported that direct extraction in Lysis Buffer (L-like method) produced a reduced number of spots and increased horizontal streaking when compared to the TP method. This observation is not in agreement with our work, which finds that protein extraction with the L method produces high spot counts and minimum horizontal streaking. The reported horizontal streaking is either due to the presence of interfering substances, which can arise from the more recalcitrant nature of the fifth maize leaves used by Wu *et al*., or from the presence of nucleic acids since the use a of nuclease is not reported [3]. Nucleic acids are known to interfere with IEF [11].

**Fig 3:**
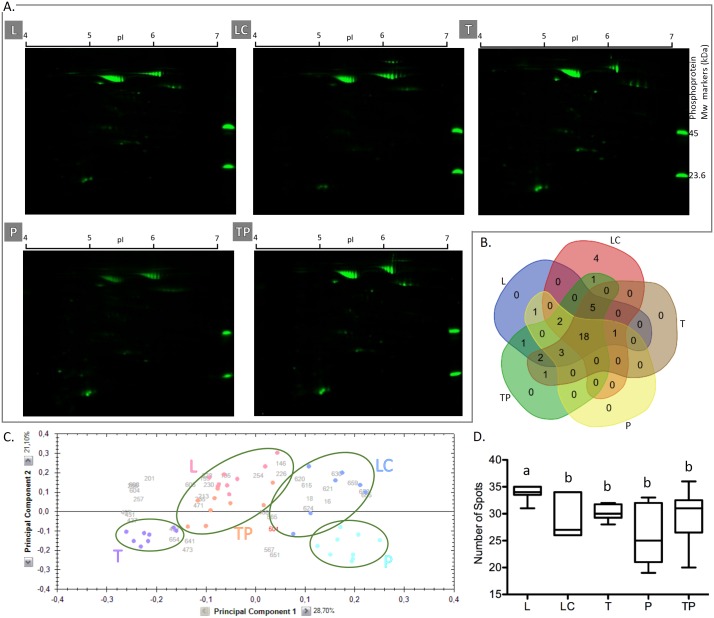
Comparative study of phosphoproteomes obtained with five different extraction methods. A: 2DE gels of protein extracts obtained with the extraction methods L—Lysis Buffer, LC—Lysis Buffer + Cleanup, T—TCA/Acetone Precipitation, P—Phenol Extraction, TP—TCA/Acetone Precipitation + Phenol Extraction stained with Pro-Q Diamond^®^ stain. B: Venn diagram summarizing the overlap of the total 39 spots detected in phosphoproteome analysis. C: Principal component analysis of the 39 spots found to be differently abundant between all five methods: L (pink); LC (blue); T (purple); P (turquoise blue); TP (orange). D. Box plot of the total number of detected spots; different letters indicate that the spot counting was found to be statistically different (p < 0.05) between the extraction methods. Phosphoprotein Mw markers: phosphoproteins molecular weight markers in kDa; pI: isoelectric point.

For the phosphoproteome analysis, 2DE gels were stained with Pro-Q Diamond phosphoprotein stain (PQD) (Fig 3) and a total number of 39 phosphoprotein spots were detected. Comparing all the methods based on the median value, the use of the L method resulted in the recovery of the highest number of PQD-stained spots (34 spots). The median value of the total number of spots detected with the methods LC, T, P, and TP was 27, 30, 25, and 31, respectively (Fig 3D). The statistical analysis revealed that the higher number of spots detected with the L method was statistically significant (p < 0.05) when compared with any of the other methods. In the phosphoproteome analysis, no other differences in the total number of spots were found to be statistically significant (Fig 3D). All 39 detected spots were found to be differently abundant (p < 0.05) within the five tested methods. The PCA of those differently abundant phosphoprotein spots (Fig 3C) shows that the five extraction methods group in a similar manner to that observed for the whole proteome analysis.

The above results provide evidence that L is the best method to study the phosphoproteome. It recovers the highest number of phosphorylated isoforms, with good total protein yield. Additionally, we found that the L method increases the abundance of the more acidic isoforms of specific proteins (see S1A Fig) that eventually are the isoforms with higher phosphorylation degree (see S1B Fig).

### Protein Identification

To assess the quality of the protein extraction obtained, we characterized the 2DE maize protein map by MALDI-TOF/TOF MS. This approach also allowed us to ensure the extraction of our proteins of interest (maize C4-photosynthesis proteins) and to provide a 2DE map of the third maize leaf.

**Fig 4:**
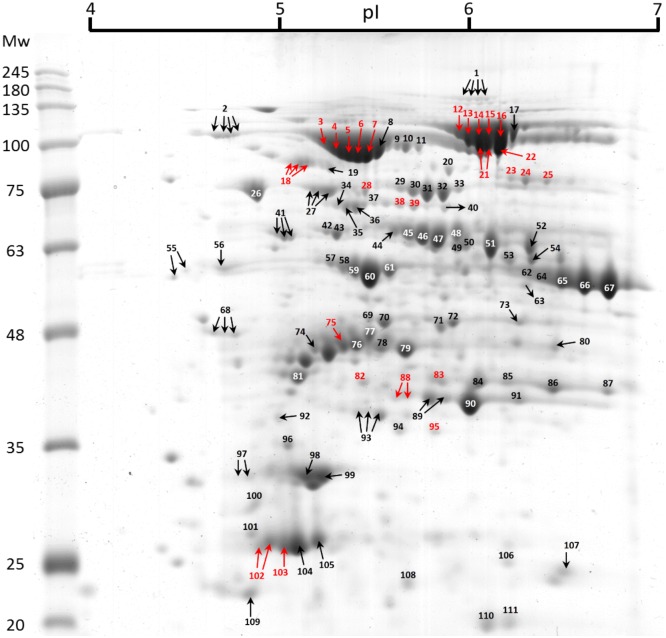
Full 2DE map with annotation of the protein spots identified by MALDI-TOF/TOF. The protein spots that co-localize with the Pro-Q Diamond^®^ signal are shown in red. Mw—molecular weight markers in kDa, pI—isoelectric point.

**Fig 5:**
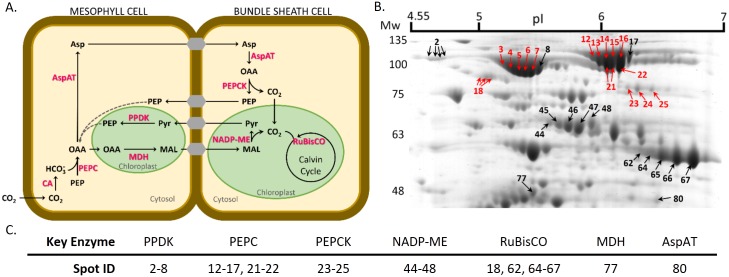
Key enzymes of the C4-photosynthetic metabolism. A. Schematic representation of the C4-photosynthetic metabolic pathways present in maize (adapted from [12]). B. and C. Spot localization in 2DE gel of the C4-photosynthesis related proteins. The abbreviations used are: Asp—aspartate, AspAT—aspartate aminotransferase; CA—carbonic anhydrase; MAL—malate; MDH—malate dehydrogenase; Mw—molecular weight markers (kDa); NADP-ME—NADP-dependent malic enzyme; OAA—oxaloacetate, PEP—phosphoenolpyruvate; PEPC—PEP carboxylase; PEPCK—PEP carboxykinase; pI—isoelectric point; PPDK—pyruvate orthophosphate dikinase; Pyr—pyruvate; and RuBisCO—ribulose-1,5-bisphosphate carboxylase/oxigenase. The protein spots that co-localize with the Pro-Q Diamond^®^ signal are shown in red.

Fig 4 shows the excised protein spots and their numbering. Table 2 lists the results of spot identification by MALDI-TOF/TOF MS (for more detailed information per spot, see S1 Table). The spots labeled in red in Fig 4 are underlined in Table 2 and are the ones that co-localized with the PQD signal. Protein identification by mass spectrometry (MS) resulted in the identification of 58 proteins, seven being key enzymes in maize C4-photosynthesis (Fig 5). Twenty-five spots observed with PQD could be co-localized with spots in the gel stained with CBB. Their identification by MS resulted in the identification of 13 putative phosphorylated proteins, four of which are key enzymes in maize C4-photosynthesis (Fig 5).

**Table 2:** Proteins identified by MALDI-TOF/TOF MS.

Spot ID	AC	Protein name	T pI	T Mw
1	P23225	Ferredoxin-dependent glutamate synthase, chloroplastic	5.70	165 204
2, 3, 4, 5, 6, 7, 8	P11155	Pyruvate, phosphate dikinase 1, chloroplastic	5.27	96 157
9, 10	Q2QVG9	Chaperone protein ClpC2, chloroplastic	5.89	96 205
11	Q7F9I1	Chaperone protein ClpC1, chloroplastic	6.14	101 738
12, 13, 14, 15, 16, 17, 21, 22	P04711	Phosphoenolpyruvate carboxylase 1	5.77	109 228
18	Q37282	Ribulose bisphosphate carboxylase large chain	6.04	52 026
19	Q9SI75	Elongation factor G, chloroplastic	5.06	77 620
20	Q2QLY5	5-methyltetrahydropteroyltriglutamate—homocysteine methyltransferase 1	5.93	84 531
23, 24, 25	Q9SLZ0	Phosphoenolpyruvate carboxykinase [ATP]	6.57	73 267
26	Q9STW6	Heat shock 70 kDa protein 6, chloroplastic	4.79	67 146
27	P11143	Heat shock 70 kDa protein	5.22	70 529
28	Q5Z974	ATP-dependent zinc metalloprotease FTSH 1, chloroplastic	5.46	71 150
29, 30, 31, 32, 33	Q7SIC9	Transketolase, chloroplastic	5.47	72 947
34, 35	Q655S1	ATP-dependent zinc metalloprotease FTSH 2, chloroplastic	5.40	69 383
36	Q7XRA1	Arginine decarboxylase 2	6.45	67 292
37, 38	P49087	V-type proton ATPase catalytic subunit A	5.88	61 912
39, 40	P93804	Phosphoglucomutase, cytoplasmic 1	5.46	63 057
41	P08823	RuBisCO large subunit-binding protein subunit alpha, chloroplastic	4.83	57 357
42	P08927	RuBisCO large subunit-binding protein subunit beta, chloroplastic	5.26	57 893
43	C0Z361	Chaperonin 60 subunit beta 3, chloroplastic	5.63	60 396
44, 45, 46, 47, 48	P16243	NADP-dependent malic enzyme, chloroplastic	5.36	63 334
49, 50, 52	P05022	ATP synthase subunit alpha, chloroplastic	5.87	55 672
51	Q6L3A1	ATP synthase subunit alpha, chloroplastic	5.87	55 716
53	P05494	ATP synthase subunit alpha, mitochondrial	5.85	55 145
54	Q41761	4-hydroxy-7-methoxy-3-oxo-3,4-dihydro-2H-1,4-benzoxazin-2-yl glucoside beta-D-glucosidase 2, chloroplastic	5.82	58 429
55	Q7XBW5	Probable plastid-lipid-associated protein 3, chloroplastic	4.13	35 103
56, 57, 58, 59, 60, 61	P00827	ATP synthase subunit beta, chloroplastic	5.31	54 007
62, 64, 65, 66, 67	P00874	Ribulose bisphosphate carboxylase large chain	6.35	52 449
63	P55229	Glucose-1-phosphate adenylyltransferase large subunit 1, chloroplastic	6.06	51 798
68	O64422	Fructose-1,6-bisphosphatase, chloroplastic	4.67	38 812
69,70	Q43467	Elongation factor Tu, chloroplastic	5.27	44 511
71	P12783	Phosphoglycerate kinase, cytosolic	5.64	42 095
72	P29409	Phosphoglycerate kinase, chloroplastic	5.29	42 600
73	Q42961	Phosphoglycerate kinase, chloroplastic	5.59	42 552
74	A2Y053	S-adenosylmethionine synthase 1	5.74	43 192
75, 76	Q9ZT00	Ribulose bisphosphate carboxylase/oxygenase activase, chloroplastic	5.21	42 538
77	P15719	Malate dehydrogenase [NADP], chloroplastic	5.00	42 534
78	P12860	Glyceraldehyde-3-phosphate dehydrogenase B, chloroplastic	5.76	39 332
79	P25462	Glutamine synthetase, chloroplastic	5.41	40 974
80	P26563	Aspartate aminotransferase P2, mitochondrial	6.23	44 640
81	P46285	Sedoheptulose-1,7-bisphosphatase, chloroplastic	6.04	42 034
82, 88, 89, 90, 91	Q40677	Fructose-bisphosphate aldolase, chloroplastic	5.35	37 983
83, 85, 86, 87	P09315	Glyceraldehyde-3-phosphate dehydrogenase A, chloroplastic	6.25	36 073
84	P0C1M0	ATP synthase subunit gamma, chloroplastic	5.66	36 080
92	B4FT40	Single myb histone 2	8.44	32 608
93, 94, 95	P41344	Ferredoxin—NADP reductase, leaf isozyme, chloroplastic	8.11	33 980
96, 101, 102	P12329	Chlorophyll a-b binding protein 1, chloroplastic	4.99	24 842
97, 100	Q01526	14-3-3-like protein GF14-12	4.75	29 617
98	Q9S841	Oxygen-evolving enhancer protein 1–2, chloroplastic	5.02	26 555
99	P23321	Oxygen-evolving enhancer protein 1–1, chloroplastic	5.94	20 197
103, 105	P27497	Chlorophyll a-b binding protein M9, chloroplastic	4.98	24 822
104	P43188	Adenylate kinase, chloroplastic	4.95	24 851
106	Q9ZTP5	Ribulose-phosphate 3-epimerase, chloroplastic	5.73	24 934
107	P12302	Oxygen-evolving enhancer protein 2, chloroplastic	5.94	20 197
108	Q32904	Chlorophyll a-b binding protein 3, chloroplastic	8.84	29 588
109	Q6ER94	2-Cys peroxiredoxin BAS1, chloroplastic	4.64	22 195
110	Q6ZBZ2	Germin-like protein 8–14	5.67	19 355
111	P06586	30S ribosomal protein S3, chloroplastic	9.76	25 900

AC—Swiss-Prot accession number; Protein name—The name provided by the database for the protein identified; T pI—calculated theoretical isoelectric point; T Mw—calculated theoretical molecular weight. Underlined Spot numbers correspond to phosphorylated proteins (labeled in red in Fig 4).

Amongst the proteins that co-localize with the PQD signal and that are not key enzymes of C4-photosynthesis, chlorophyll *a*-*b* binding proteins 1 and M9 (spots 102 and 103, respectively) are the only ones with phosphorylation sites annotated in the Swiss-Prot database (T33 and T35, respectively). Additionally, some large-scale studies of maize phosphoproteomics report phosphorylated residues in several proteins that we found as co-localizing with the PQD signal [13, 14].

In the work of Ning *et al*. [14], an additional phosphorylated residue (S47) is reported for the protein previously annotated as “phosphorylated chlorophyll a-b binding protein M9” (spot 103). A total of 3 phosphorylation sites (T189, S191, and S124) were reported for phosphoglucomutase (spot 39) in the two large-scale analysis mentioned above [13, 14]. Ning *et al*. [14] also report four phosphorylated residues (T310, T311, T316, and S317) for V-type proton ATPase (spot 38), one for fructose-bisphosphate aldolase (S334; spot 82, and 88) and two for glyceraldehyde-3-phosphate dehydrogenase (S92, and S93; spot 83).

For the remaining proteins that co-localize with PQD signal and are not key enzymes in C4-photosynthesis, we could not find reports describing phosphorylation sites in maize. However, phosphorylation sites were reported for RuBisCO Activase (spot 75) in *Arabidopsis thaliana* [15]. The coincidence of our putatively identified phosphorylated proteins with the reports in maize and Arabidopsis is a strong indication that the method described here is good at maintaining phosphorylation sites. Still, there are no comprehensive reports on the phosphoproteome of photosynthetic maize C4-leaves, and these will be crucial to catalog and study phosphorylation of maize proteins of the C4-metabolism.

### C4-photosynthesis key enzymes

A schematic representation of the maize C4-metabolism and its key enzymes are provided in Fig 5A. Fig 5B shows the 2DE map region that contains all the spots identified as key enzymes of the C4-photosynthesis (annotated in Fig 5C and Table 2). It is noteworthy that most proteins of this photosynthetic system have a pI between pH 4 and 7, thus the choice of the IPG strip pH range.

When comparing the maps obtained with the five tested methods, it is clear that different strategies lead to the recovery of a different number of phosphoenolpyruvate carboxylase (PEPC) isoforms (spots 12–17, and 21 and 22). The protein extracts obtained with the T method [Figs 2A(T) and 3A(T)] only revealed three isoforms of this protein, whereas the other methods allowed recovery of up to six isoforms. The differences in protein recovery probably result from variations in protein re-solubilization, occurring after precipitation steps during sample preparation.

Regarding the results obtained with PQD staining, it was possible to identify several putative phosphorylated isoforms of PEPC, pyruvate orthophosphate dikinase (PPDK), phosphoenolpyruvate carboxykinase (PEPCK), and the ribulose bisphosphate carboxylase/oxygenase (RuBisCO) large subunit. The available information on the Swiss-Prot database indicates one phosphorylation site (S15) annotated for PEPC and four for PPDK (T309, S506, T527, and S528). Other phosphorylated residues were described for both proteins: S136 and T138 for PEPC [13, 14]; and T462 and T463 for PPDK [14]. Moreover, phosphorylation was already described to regulate the activity of those two C4-photosynthesis enzymes. In PEPC, the phosphorylation of a serine residue on its N-terminal (S15 in maize) is responsible for tuning the protein activity by decreasing PEPC sensitivity to malate inhibition, in light conditions [16]. In the case of PPDK, the phosphorylation of a threonine residue in the central domain of the protein (T527 in maize) negatively impacts on enzyme activity during the night [17]. Despite the fact that there are no phosphorylation sites described for PEPCK or RuBisCO large subunit in the UniProt database, some recent publications reported several phosphorylation sites. For PEPCK, four phosphorylated residues (S55, T58, T59, and T120) were reported in 2014 [18], and an additional one (S119) was published in 2015 [14]. For RuBisCO large subunit, Bi *et al*. [13] reported the identification of a phosphorylation site in *Tetraselmis marina* while analyzing maize tissues. Additionally, in *Arabidopsis thaliana* several phosphorylation sites have been reported for RuBisCO large subunit [19].

Our data suggest the existence of more than one phosphorylation site in the identified phosphorylated proteins since several isoforms could be detected. Nevertheless, other PTMs that also result in a pH shift towards the acidic pI (*e*.*g*. acetylation) cannot be excluded and, most certainly, also contribute to the existence of several protein isoforms. Further gel-based studies will be necessary to assign specific combinations of phosphorylation sites to each specific protein isoform. Also, our results cannot exclude the existence of other phosphorylated proteins that either were lost during purification or are present below our detection threshold. Therefore, different processing methodologies (*e*.*g*. phosphoprotein enrichment) may have to be incorporated into the protocol for a comprehensive characterization of maize phosphoproteins.

## Conclusions

In this work, we showed that all the tested methods are suitable for protein extraction for a 2DE-MS approach to properly separate and identify proteins. Importantly, all the extraction methods described in this study can be used in LC-MS/MS workflows. However, limitations associated with the extraction methods will remain.

We clearly show that different protein extraction methods generate different protein-profiles. Amongst the five tested methods we conclude that the extraction in Lysis Buffer (L method) is the most appropriated for phosphoproteome analysis, because it results in: (i) the extraction of the most complete phosphoproteome amongst tested methods; (ii) good proteome coverage; (iii) high protein yield; (iv) high reproducibility; and (v) little protein degradation. The L method performance probably results from its apparent ability to extract the most acidic isoforms of proteins. We also determined that the L method is adequate for the recovery of some key enzymes of the C4-photosynthesis, namely PEPC.

Eventually, for phosphoproteome studies in recalcitrant tissues, the TP method may be a more suitable method since it significantly increases the quality of the extraction by removing interfering substances. However, as we show here, the TP method may compromise the number of recovered phosphorylated isoforms. In summary, the L method is a fast and easy-to-perform extraction method for use in phosphoproteomics studies of plant tissues with reduced recalcitrance.

## Supporting Information

S1 Fig Abundance of acidic isoforms. The standardized normalized volume of the spots corresponding to the acidic isoforms observed with whole proteome staining (CBB, A: all spots numbered in C) and the same isoforms found with phosphoproteome staining (PQD, B: all spots numbered in red in C). In A and B, each dot (square) represents the abundance of a spot in a gel. Squares linked by the line show the abundance of the same spot in the different gels prepared in this work. In C the 2DE gel stained with CBB shows the isoforms with abundance indicated in A, numbered according to Table 2. Numbers in red indicate the isoforms that were also detectable with PQD staining and that have their abundance presented in B.
(TIF)

S1 Table Spots identification by MALDI-TOF/TOF MS. AC—Swiss-Prot accession number, Protein name—The name provided by the database for the protein identified, Search—Indication of search parameters used in the spot identification according to the iterative search described in material and methods section (50z –taxonomic restriction for *Zea mays* with 50 ppm error allowed, threshold: 41; 50v –taxonomic restriction for *Viridiplantae* with 50 ppm error allowed, threshold: 58; 100a –no taxonomic restriction with 100 ppm error allowed, threshold: 70).
(XLSX)
