# Supplementary figures and images for: Selection of an Appropriate Protein Extraction Method to Study the Phosphoproteome of Maize Photosynthetic Tissue
Source: PLoS One. 2016 Oct 11;11(10):e0164387. doi: 10.1371/journal.pone.0164387 (PMC5058499; doi:10.1371/journal.pone.0164387)

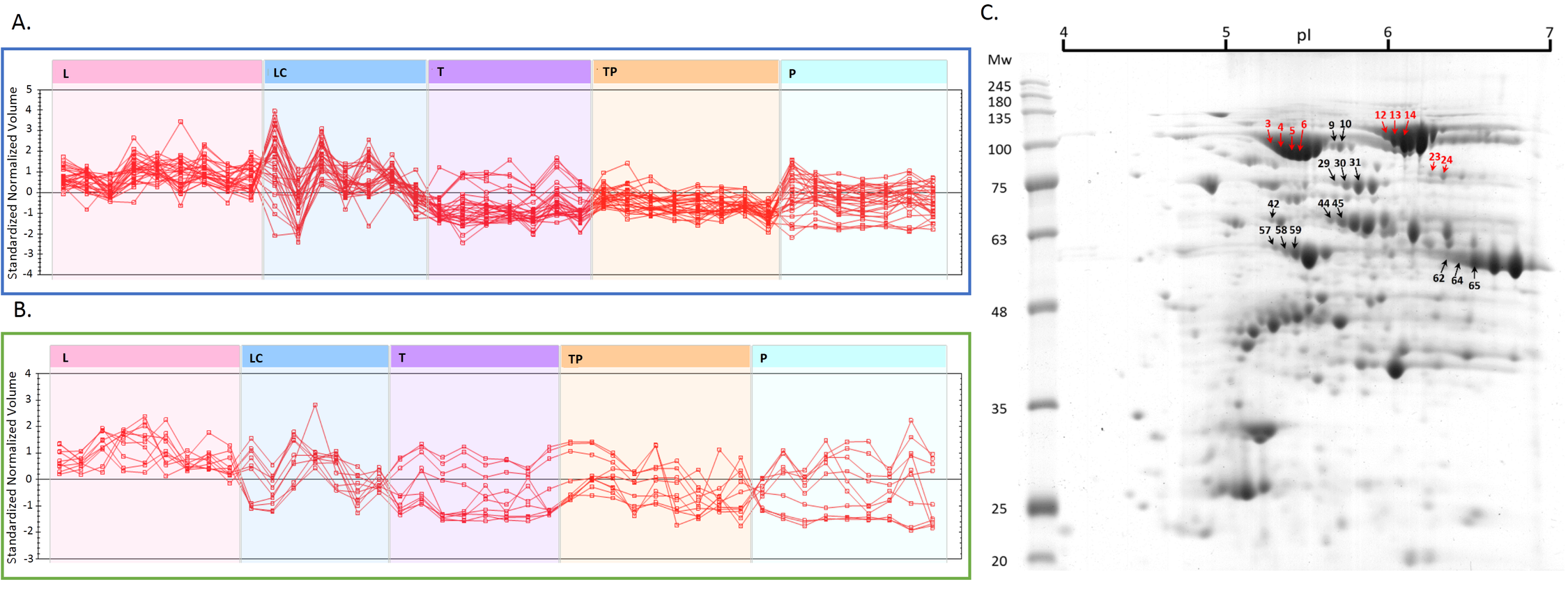

Supplement: S1 Fig — The standardized normalized volume of the spots corresponding to the acidic isoforms observed with whole proteome staining (CBB, A: all spots numbered in C) and the same isoforms found with phosphoproteome staining (PQD, B: all spots numbered in red in C). In A and B, each dot (square) represents the abundance of a spot in a gel. Squares linked by the line show the abundance of the same spot in the different gels prepared in this work. In C the 2DE gel stained with CBB shows the isoforms with abundance indicated in A, numbered according to Table 2. Numbers in red indicate the isoforms that were also detectable with PQD staining and that have their abundance presented in B. (TIF) [file pone.0164387.s001.tif]
